# Parent Perspectives Towards Genetic and Epigenetic Testing for Autism Spectrum Disorder

**DOI:** 10.1007/s10803-019-03990-6

**Published:** 2019-03-22

**Authors:** Kayla E. Wagner, Jennifer B. McCormick, Sarah Barns, Molly Carney, Frank A. Middleton, Steven D. Hicks

**Affiliations:** 1 Department of Pediatrics, Penn State College of Medicine, 500 University Drive, Mail Code HS83, Hershey, USA; 2 Quadrant Biosciences Inc., Syracuse, USA; 3 Departments of Neuroscience & Physiology, Psychiatry, Biochemistry & Molecular Biology, Pediatrics, State University of New York Upstate Medical University, Syracuse, USA; 4 Department of Humanities, Penn State College of Medicine, 500 University Drive Mail Code H134, Hershey, USA

**Keywords:** Autism, Parent perspectives, Epigenetics, Diagnosis, Bioethics

## Abstract

Examining community views on genetic/epigenetic research allows collaborative technology development. Parent perspectives toward genetic/epigenetic testing for autism spectrum disorder (ASD) are not well-studied. Parents of children with ASD (n = 131), non-ASD developmental delay (n = 39), and typical development (n = 74) completed surveys assessing genetic/epigenetic knowledge, genetic/epigenetic concerns, motives for research participation, and attitudes/preferences toward ASD testing. Most parents (96%) were interested in saliva-based molecular testing for ASD. Some had concerns about privacy (14%) and insurance-status (10%). None (0%) doubted scientific evidence behind genetic/epigenetic testing. Most reported familiarity with genetics (88%), but few understood differences from epigenetics (19%). Child developmental status impacted insurance concerns (p = 0.01). There is broad parent interest in a genetic/epigenetic test for ASD. It will be crucial to carefully consider and address bioethical issues surrounding this sensitive topic while developing such technology.

## Introduction

Autism spectrum disorder (ASD) encompasses a set of heterogeneous conditions, characterized by atypical development in social communication and interaction, as well as restricted and repetitive patterns of behaviors and interests (Lai et al. 2014). ASD is a highly genetic condition with numerous known genetic risk factors (Anagnostou et al. 2014; Carter and Scherer 2013). In addition, gene-environment interactions are believed to have an important role in the etiology of the disorder (Lai et al. 2014). Behavioral tests are the mainstay for ASD diagnosis, but genetic testing, such as microarray analysis, can be used to identify known genetic disorders that may provide a conceptual framework for symptom presentation. The 2007 American Academy of Pediatrics (AAP) guidelines, followed by clinicians in the United States, as well as many international physicians, recommend the use of genetic testing as part of a comprehensive ASD evaluation (Johnson and Myers 2007). Increasingly, other genetic technologies are being used, including whole exome (or genome) sequencing and epigenetic testing.

Epigenetics refers to changes in gene expression, where the changes are not due to modification of the actual DNA sequence, but instead result from modifications that regulate DNA structure and expression. Epigenetic factors can fluctuate in response to the internal (cellular) and external environments (Rothstein et al. 2009), which may allow them to confer information about gene-environment interactions in conditions such as ASD.

Examining the influence of epigenetics on gene expression has helped increase our understanding of neurological development and function. Epigenetics has been implicated in various psychological conditions including ASD (Landgrave-Gómez et al. 2015). Continued research on both genetic and epigenetic risk factors for ASD will help improve our understanding of this complex disorder, potentially leading to improved clinical diagnostics (Loke et al. 2015).

To date, there is limited research investigating parental perceptions of genetic research and genetic testing for ASD. Studies previously examined parent perceptions of the utility of test results, how test results influence parents’ beliefs about ASD etiology and prognosis, and whether parents are supportive of genetic research (Fischbach et al. 2016; Johannessen et al. 2016; Reiff et al. 2017, 2015; Xu et al. 2018). Numerous other studies examined the general public’s willingness to undergo genetic testing or participate in genetic research broadly, as well as basic genetic literacy (Chokoshvili et al. 2017; Henneman et al. 2012; Lee et al. 2018). While support for research is generally high (Ahram et al. 2014; Biesecker et al. 2009; Facio et al. 2011; Gollust et al. 2012; Harris et al. 2012; Ludman et al. 2010; Olson et al. 2013; Trinidad et al. 2010), there is a broad range of public awareness and understanding regarding genetic principles. Generally, the hereditary aspect of genetics is first mentioned by the public when examining genetics knowledge (Lemke et al. 2010; Molster et al. 2009; Oberg et al. 2015). Recent literature suggests that individuals are familiar with terminology surrounding genetics but may not understand basic concepts (Catz et al. 2005; Lanie et al. 2004; Lea et al. 2011; Miller 2004). Findings have also demonstrated a general understanding among the public of concepts related to reported genetic association vs. causation (Bates et al. 2003; Goodacre et al. 2005; Miller 2004).

Previous studies largely focused on understanding the perspectives of research participants and patients related to genetic and genomic testing in the context of their ethical, legal, policy, and social implications (ELSI). Surveys, interviews, and focus groups reveal several lingering concerns, such as (1) who will have access to genetic test information, especially when the information is in electronic medical records (Beskow and Dean 2008; Hull et al. 2008; Kettis-Lindblad et al. 2006; McGuire et al. 2008; Trinidad et al. 2010), (2) will genetic research data be shared with outside investigators (Abraham et al. 2014; Kaufman et al. 2009; Ludman et al. 2010; Rahm et al. 2013), and (3) will individuals have access to their own genetic research data (Godard et al. 2007; Haga and Zhao 2013; Ormond et al. 2009). These studies suggest that privacy and confidentiality are particularly important in the context of discrimination by insurers and employers who may have access to genetic information (Wong et al. 2004). While many researchers have examined the ELSI of genetic and genomic research and testing, there is a lack of research focusing on the implications of epigenetic testing. A recent study examining parental attitudes and beliefs associated with epigenetic testing revealed major gaps in overall knowledge of epigenetics (Sapp et al. 2014).

Understanding how parents perceive and understand epigenetic research is an important topic, given the divide that has existed between the goals of ASD researchers and the ASD community (Pellicano and Stears 2011). Current diagnostic evaluations for ASD rely on objective behavioral assessments, but recent advances in genetic/epigenetic research make the possibility of a molecular diagnostic test increasingly likely (Hicks et al. 2016, 2018; Hicks and Middleton 2016).

Recently, we published studies that demonstrate the utility of RNA sequencing technology (non-coding RNA) to identify children with ASD (Hicks et al. 2016, 2018). In conjunction, we conducted a survey with parents of children participating in these studies. The goals of this survey were to determine parents’ understanding of genetics and epigenetics, parental motives for enrolling their child in an epigenetic study, and concerns parents might have about epigenetic testing. We explored the influence of child developmental status on each of these parental views. Based on previous studies examining motives for participation in genetic/epigenetic research, we hypothesized that a desire to help children would be the strongest driver of participation, and that parents would generally desire return of full epigenetic results, regardless of baseline knowledge of epigenetics or the implications of the results on child health. We also posited that child developmental status (ASD, typical development, or non-ASD developmental delay) might impact parental views.

## Methods

### Ethics Statement

This study was approved by the Institutional Review Boards (IRB) at the State University of New York (SUNY) Upstate Medical University and the Penn State College of Medicine. Informed consent was obtained by a trained professional for all participants.

### Participants

A 16-question survey was developed to collect data on parental perspectives toward genetic and epigenetic testing for ASD. The 16-question survey was modeled from previous studies investigating attitudes toward genetic testing (Gollust et al. 2012; Johannessen et al. 2016; Reiff et al. 2015; Xu et al. 2018) in consultation with pediatric physicians and genetics bioethics faculty at the Penn State College of Medicine. Though this is the first study (to our knowledge) to investigate parental perspectives toward epigenetic testing in ASD, standard principles of survey development have been applied to ensure external psychometric validity (Scheaffer et al. 2011). Parents of children participating in a National Institutes of Health (NIH) study of salivary biomarkers for ASD (Hicks et al. 2018) were included in the study. Recruitment involved dissemination of flyers and brochures, as well as targeted recruitment at previously scheduled clinical encounters affiliated with SUNY Upstate Medical University (Upstate New York) and Penn State College of Medicine (Central Pennsylvania). Inclusion criteria for the parent study included having a child between ages 2–6 years with a diagnosis of ASD, non-ASD developmental delay (DD), or typical development (TD). The ASD group included parents of children with a Diagnostic and Statistical Manual (DSM-5) clinical diagnosis of ASD. Parents of children with delays in speech development, fine/gross motor development, or intellectual disability, who did not meet criteria for ASD, were placed in the non-ASD DD group. The TD group consisted of parents whose children displayed typical developmental milestones at their most recent well child surveillance visit. Children were placed in the ASD group following a diagnostic assessment by a trained clinician (e.g., developmental pediatrician or developmental psychologist) using DSM-5 criteria. Children in the TD group were enrolled following confirmation of typical developmental milestones at a yearly well-child visit. Children in the non-ASD DD group were differentiated from those with ASD using a negative MCHAT-R and/or a diagnostic evaluation with standardized measures (e.g. Autism Diagnostic Observation Schedule—2nd Edition, Autism Diagnostic Interview—Revised). Exclusion criteria for all groups included primary language other than English, children with gastrostomy tube dependence, and children who were wards of the state. Parents were excluded from the TD group if their child had a chronic medical condition requiring daily medication or pediatric specialist care.

### Data Collection

The survey for this study was administered to a single parent, in a private room, by a trained research professional. Parents were asked to review a one-page handout with IRB-approved information about genetics and epigenetics before completing the 16-question survey. Survey questions were multiple-choice, with the option to choose one or more answers for each question. Questions examining parental perspectives on genetic/epigenetic testing were organized into six themes: (1) reasons for participating in the epigenetic study; (2) prior knowledge of genetics/epigenetics, and the source of this information; (3) overall interest in genetic/epigenetic testing for ASD; (4) concerns about genetic/epigenetic testing for ASD; (5) preferences about the approach for genetic/epigenetic testing (including age of administration and biofluid of choice); and (6) extent of results to be returned.

### Data Analysis

Data was prepared for analysis using SPSS software. Mean parental age, and proportions of parental sex, race, ethnicity, and education level were determined for each developmental group (ASD, DD, and TD). On each of the 16 survey questions, the proportion of positive respondents was determined for each potential response. Student’s two-tailed t-tests examined mean differences in responses between ASD, DD, and TD groups, as well as differences in socio-demographic factors (parent age, gender, race, ethnicity, education level). Lastly, multifactorial general linear model analysis of variance (GLM ANOVA) was conducted to examine all possible contributing factors and confounding variables for responses to each of the 16 survey items. In each model, we examined the impact of parent age, race, gender, education level, child diagnosis, previous knowledge of genetics/epigenetics and source of information about genetics/epigenetics (e.g., teacher, doctor, internet, books, news) on the response items.

## Results

### Participant Characteristics

A total of 244 parents (mean age 35, 86.9% female, 76.2% Caucasian; Table 1) were included in the analyses. Among the parents in the current sample, 54% (131/244) had a child with ASD, 16% (39/244) had a child with DD, and 30% (74/244) had a child with TD. Children included in the study ranged from 19 to 135 months old (mean age 52 months, SD = 17.6) with 78% male (189/241) and 22% (52/241) female. As shown in Table 1, parents of children with ASD did not differ from DD or TD groups in age, sex, ethnicity, race or education (all p > 0.05).

**Table 1:** Participant data

	All parents *n* = 244	ASD parents *n* = 131	DD parents *n* = 39	TD parents *n* = 74	p Value
Parent age, years (SD)	34.7 (7.97)	35.6 (8.38)	33.1 (7.29)	34.0 (7.44)	0.14
Parent sex, # female (%)	212 (86.9)	112 (85.5)	33 (84.6)	67 (90.5)	0.53
*Race*					
Caucasian, # (%)	186 (76.2)	96 (73.3)	29 (74.4)	61 (82.4)	0.32
African American, # (%)	3 (1.2)	2 (1.5)	1 (2.6)	0	0.46
Asian, # (%)	2 (0.8)	2 (1.5)	0	0	0.42
Other, # (%)	2 (0.8)	2 (1.5)	0	0	0.42
Unknown, # (%)	51 (21.0)	29 (22.2)	9 (23.0)	13 (17.6)	
Ethnicity, # (% Hispanic)	18 (7.4)	13 (9.9)	2 (5.1)	3 (4.1)	0.26
*Education*					
Less than high school, # (%)	13 (5.3)	10 (7.6)	1 (2.6)	2 (2.7)	0.23
High school diploma, # (%)	44 (18.0)	19 (14.5)	9 (23.1)	16 (21.6)	0.30
Some college, # (%)	44 (18.0)	23 (17.6)	9 (23.1)	12 (16.2)	0.66
College diploma, # (%)	70 (28.7)	36 (27.5)	13 (33.3)	21 (28.4)	0.78
Graduate degree, # (%)	69 (28.3)	39 (29.8)	7 (17.9)	23 (31.1)	0.29
Unknown, # (%)	4 (1.7)	4 (3.0)	0	0	

### Knowledge of and Source of Information About Genetics and Epigenetics

After reading a brief informational handout, parents were asked the following question about the difference between genetics and epigenetics:

Which of the following is true about genetics and epigenetics (circle all that apply)? (A) Both genetics and epigenetics measure my DNA sequence (B) Epigenetics look at the influence of environmental factors on genetic expression (C) We still do not know if any epigenetic changes cause disease (D) Epigenetic information can be used to clone cells.

**Fig. 1:**
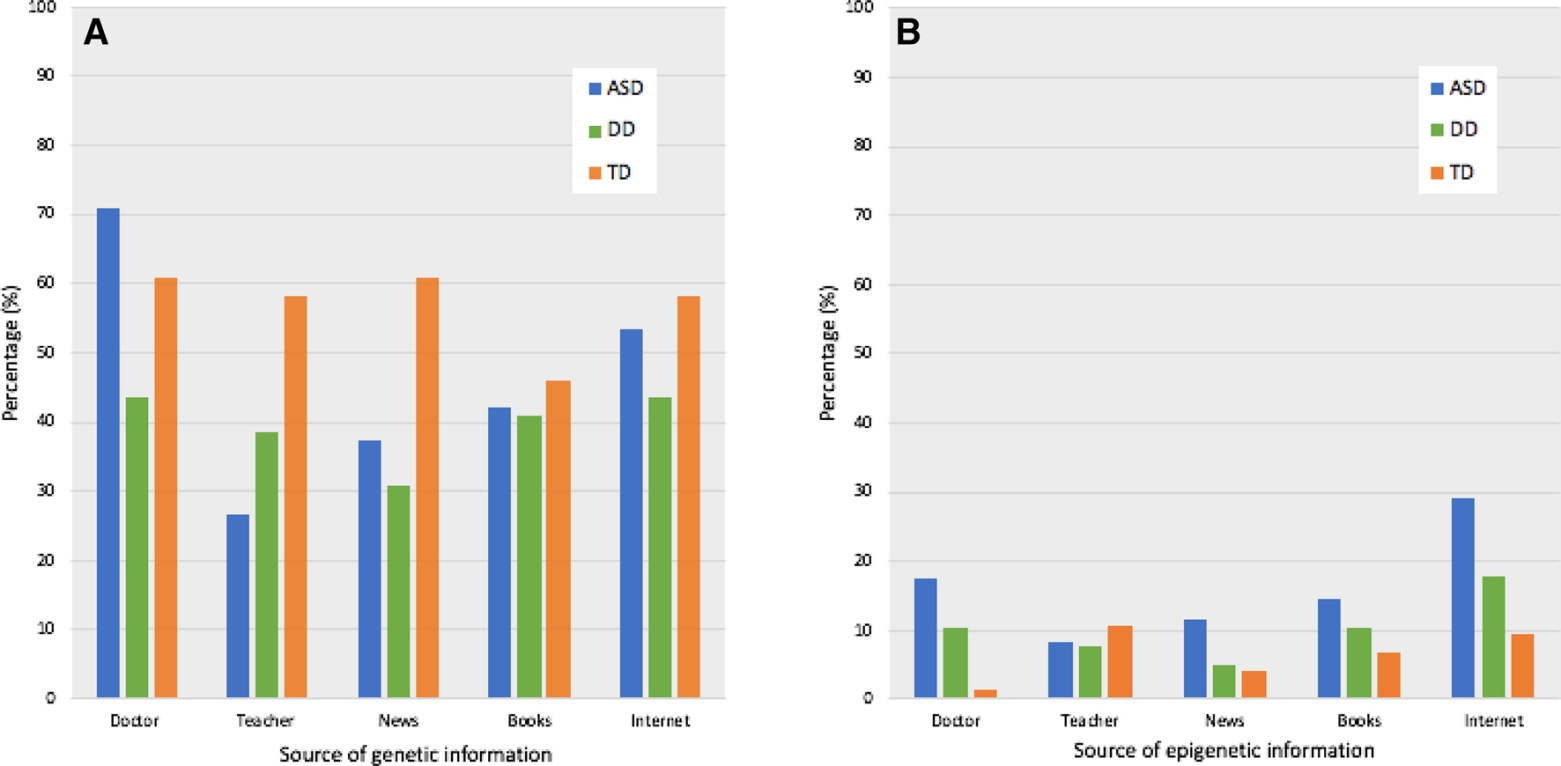
Sources of information about genetics/epigenetics. (**A**) Most parents discussed genetics with a doctor (61.5%, 150/244), while 53.3% (130/244) cited the internet as a source of genetic information, 43.4% (106/244) read about genetics in books, and 38.1% (93/244) learned about genetics from a teacher. (**B**) Among all parents, 11.5% (28/244) heard about epigenetics from a doctor, 21.3% (52/244) used the internet for epigenetic information, 8.2% (20/244) read about epigenetics in books, and 9.0% (22/244) learned about epigenetics from a teacher. Note that parents could select more than one response

Only 19% (19/98) of parents accurately responded, despite the fact that most parents (88.5%, 216/244) indicated they had some previous knowledge of genetics. Among all parents, 61.5% (150/244) had previously discussed genetic information with a doctor (Fig. 1A), while 53.3% (130/244) used the internet as a source of information, 43.4% (106/244) read about genetics in books, and 38.1% (93/244) learned about genetics from a teacher. Fewer parents indicated that they had previous knowledge about epigenetics (35.7%, 87/244). Overall, 21.3% (52/244) cited using the internet to learn about epigenetics, 11.5% (28/244) consulted with a doctor, 9.0% (22/244) learned about epigenetics from a teacher, and 8.2% (20/244) read about epigenetics in books (Fig. 1B). More parents of children with ASD had awareness of genetic and epigenetic information (91.6%, 120/131) than parents of DD (79.5%, 31/39) or TD (89.2%, 66/74) children. Parents of children with ASD were more likely to have discussed genetics with a doctor than TD parents (p = 0.01), but not DD parents (p = 0.36). Epigenetic information was also more likely to be discussed with doctors by ASD parents than TD parents (p < 0.001), but not DD parents (p = 0.28). TD parents more often used the news (p < 0.001) or consulting a teacher (p < 0.001) than the other groups to learn about genetics. DD parents read about epigenetics on the internet more than TD parents (p = 0.001).

### Concerns About Genetic/Epigenetic Testing for ASD

**Fig. 2:**
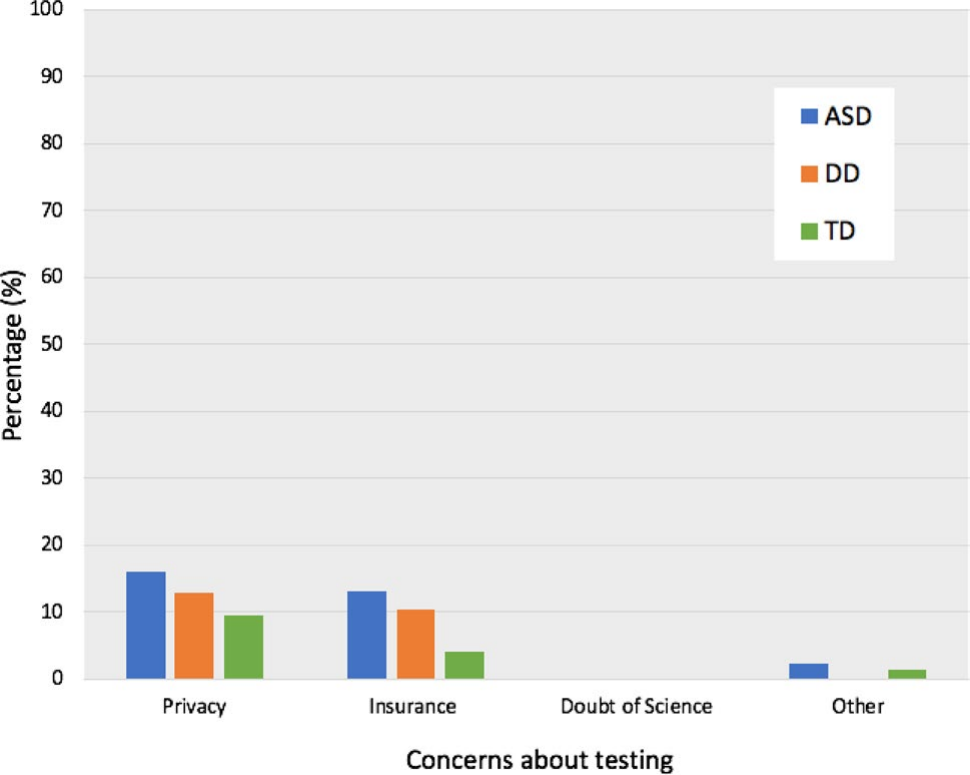
Concerns about genetic/epigenetic testing. Parents in all groups had few concerns about potential effects of testing on privacy (14%, 33/244) or insurance (10%, 24/244). No parents cited concerns regarding a lack of scientific evidence for genetic/epigenetic testing (0%, 0/244). Among all parents, 2% (4/244) cited “other” concerns, unspecified. Note that parents could select more than one response

Parental concerns about privacy, insurance, and scientific evidence related to genetic/epigenetic testing were explored (Fig. 2). Overall, few parents had concerns about the potential effect of genetic, or epigenetic testing on insurance (10%, 24/244), or privacy (14%, 33/244). There were no parents (0%, 0/244) concerned about a lack of scientific evidence supporting genetic and epigenetics. More ASD parents (13%, 17/131) than TD parents (4%, 3/74) reported concerns that genetic/epigenetic testing could affect insurance status (p = 0.04), but there were no differences between groups in privacy concerns (ASD vs. DD, p = 0.65; ASD vs. TD, p = 0.20). GLM ANOVA results revealed multiple effects of child diagnosis (F(2,243) = 4.52, p = 0.01), learning about epigenetics in books (F(1,243) = 4.83, p = 0.03) and parent race (F(5,243) = 3.06, p = 0.01) on insurance concerns, with more Asian, Hispanic and “other” parents reporting more insurance concerns than African American and Caucasian parents.

### Interest in Genetic/Epigenetic Testing for ASD

There was a positive attitude among parents toward genetic testing for ASD. Nearly all parents (96%, 235/244) indicated that if there were genetic testing for ASD, they were interested in learning results about their child’s risk for ASD. This was true regardless of whether their child had ASD (98%, 128/131), DD (100%, 39/39; p = 0.34), or TD (92%, 68/74; p = 0.05). GLM ANOVA analyses revealed a univariate effect of diagnosis on interest in genetic testing, while controlling for the effects of parent age, race, gender, and education level, F(2,243) = 4.77, p < 0.001. A similar proportion of parents were interested in epigenetic testing for ASD (95%, 232/244). This finding was similar among ASD (95%, 125/131), DD (100%, 39/39; p = 0.18), and TD parents (92%, 68/74; p = 0.31). Multiple effects of diagnosis (F(2,243) = 4.22, p = 0.02), previously learning about epigenetics from a teacher (F(2,243) = 8.14, p < 0.001), or from a doctor (F(2,243) = 5.65, p = 0.02), and previous knowledge of epigenetics (F(2,243) = 4.59, p = 0.03) was found for parent responses indicating interest in epigenetic testing for ASD.

### Interest in Learning Other Results from Testing

**Fig. 3:**
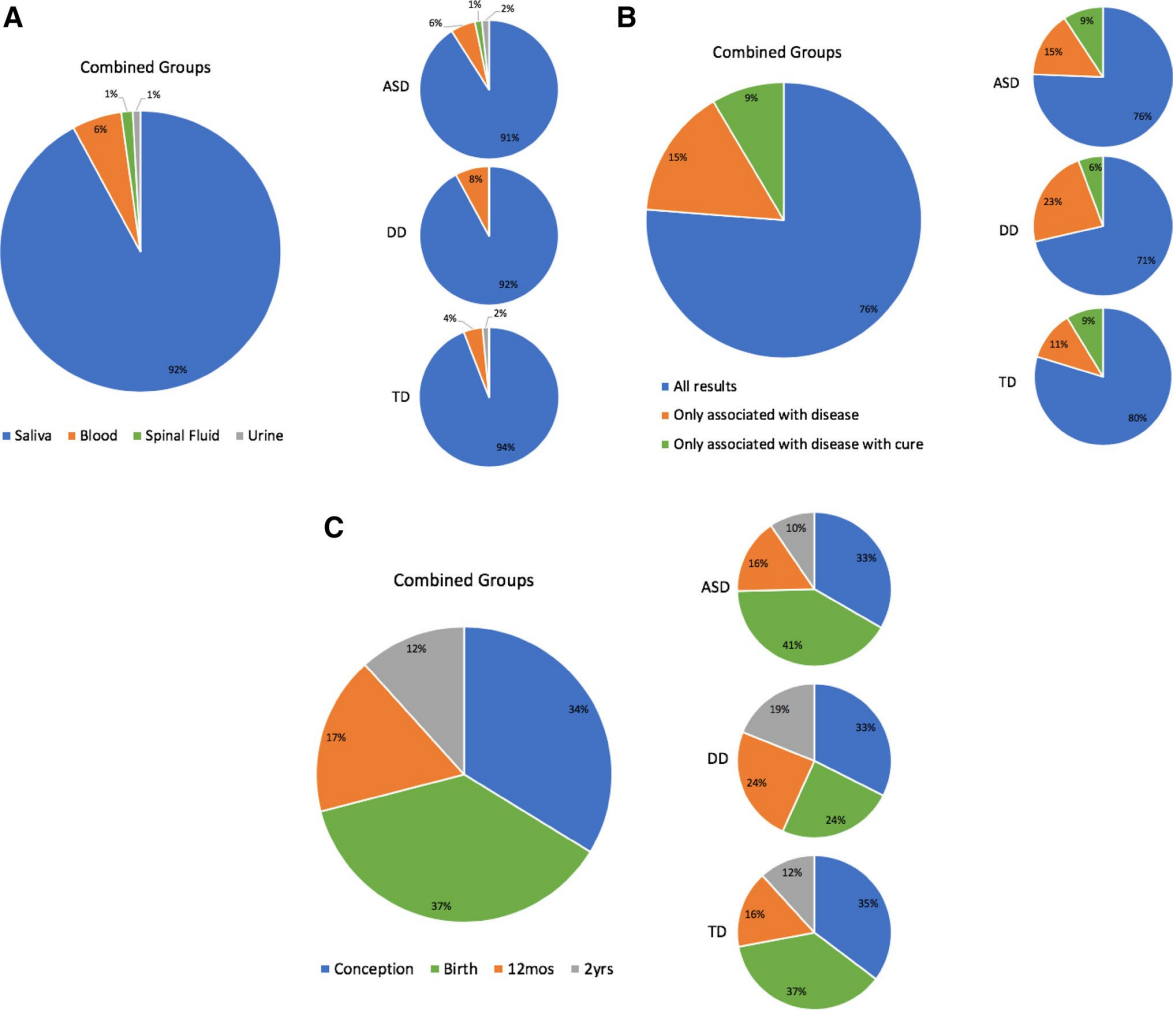
Preferences about ASD test characteristics. (**A**) The majority of parents preferred saliva (92%, 210/228), compared to blood (6%, 13/228), spinal fluid (1%, 3/228), and urine (1%, 2/228). (**B**) Parental preferences regarding the extent of genetic/epigenetic results to be returned indicated that 76.0% (170/223) of parents would like to receive all epigenetic testing results regardless of clinical ramifications, 15% (34/223) of parents preferred to receive results that showed epigenetic changes associated with a disease, while 9% (19/223) preferred to receive only epigenetic results associated with treatable disease states. (**C**) The majority of parents indicated a preference to obtain results of a test for ASD for their child either at conception (34%, 78/231) or at birth (37%, 86/231). 17% (40/231) preferred results at 12 months, while 12% (27/231) chose to receive them when their child was 2 years of age

In addition to genetic/epigenetic results conferring ASD risk, parents were asked about the extent of additional results they preferred to have returned. The majority of parents (76.0%, 170/223) indicated that they were interested in obtaining all epigenetic/genetic results, regardless of whether they were implicated in health and disease (Fig. 3B). This finding did not differ when comparing ASD parents to DD (p = 0.62) or TD parents (p = 0.52); or when comparing DD parents to TD parents (p = 0.35). Child diagnosis also had no significant impact on the overall model, (F(2,243) = 0.13, p = 0.88). There was a significant univariate effect of race on responses indicating interest in all results (F(5,243) = 2.63, p = 0.02), with more African American parents showing an interest in only results associated with a disease than the other groups. Interestingly, there was also a univariate effect of education level on responses that indicated interest in only results associated with a disease (F(1,243) = 6.71, p = 0.01).

### Preferred Developmental Time period for Result

The majority of parents (71%, 164/231) desired results of a genetic/epigenetic test for ASD when their child was 12 months of age or younger (Fig. 3C). Over half of parents were interested in receiving results at conception (34%, 78/231) or at birth (37%, 86/231), while fewer requested results at 12 months (17%, 40/231) or at 2 years of age (12%, 27/231). There was no difference between the proportion of parents who preferred genetic/epigenetic testing at conception compared to at birth (p = 0.44). Examining the impact of their child’s developmental status on timing of results showed no differences between groups for those who preferred results at conception (ASD vs. DD, p = 0.92; ASD vs. TD, p = 0.78; TD vs. DD, p = 0.77; F(2,243) = 0.07, p = 0.93) and those who preferred results at birth (ASD vs. DD, p = 0.06; ASD vs. TD, p = 0.54; TD vs. DD, p = 0.20; F(2,243) = 2.61, p = 0.08). Parent education level appeared to be linked to interest in testing at birth (F(1,243) = 4.32, p = 0.04) and at 12 months (F(1,243) = 6.09, p = 0.01).

### Reasons for Participating in the Study

**Fig. 4:**
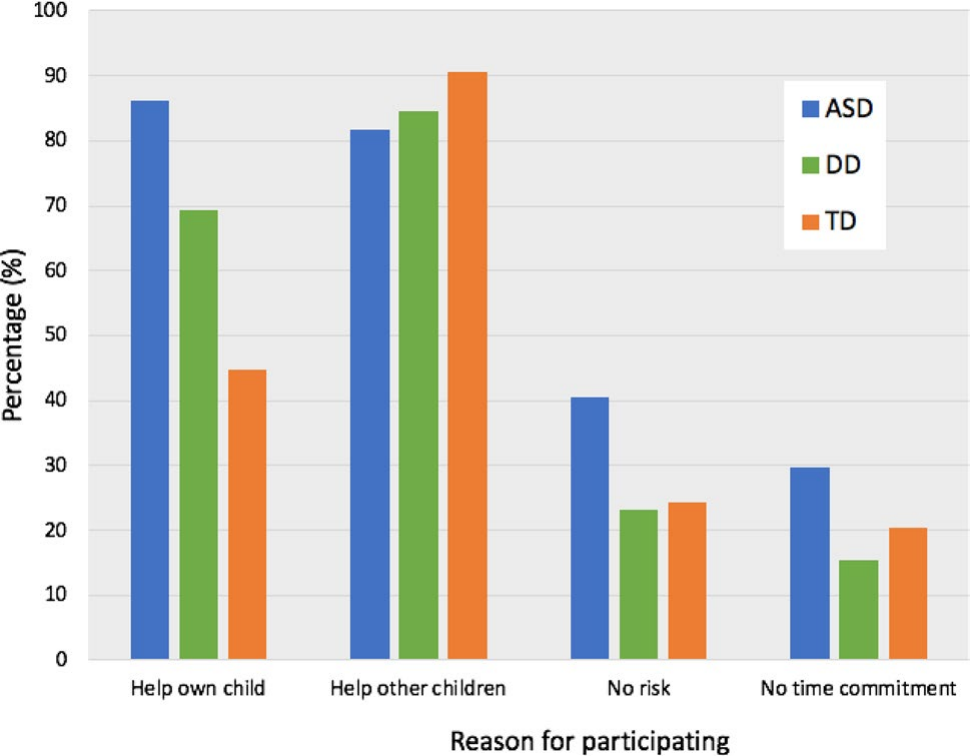
Reasons for participation in an epigenetic study of ASD. The most common responses among all parents were “helping other children in the future” (85%, 207/244), compared to helping their own child (71%, 173/244), the low risk associated with study participation (33%, 80/244), and a minimal time commitment (25%, 60/244). Note that parents could select more than one response

Parents were asked to indicate motivators for participation in the current epigenetic study for ASD (Fig. 4). Altruism or “helping other children in the future” was the most commonly endorsed factor among all parents (85%, 207/244), compared to helping their own child (71%, 173/244), low risk of study participation (33%, 80/244), and minimal time commitment (25%, 60/244). ASD parents noted personal value in the study, with a higher proportion hoping to help their own children than both DD (p = 0.01) and TD parents (p < 0.001). Parents in the DD group also indicated an interest in helping their own children more frequently than parents of TD children (p = 0.01). A multifactorial GLM ANOVA encompassing parent age, race, gender, and education level, strongly supported this finding, indicating a significant univariate effect of child diagnosis on responses indicating interest in helping their own children, F(2,243) = 20.64, p < 0.001, with most attributable variance in the model (r^2^ = 0.18) accounted for by diagnosis (r^2^ = 0.15). In addition, the majority of parents (92%, 210/228; Fig. 3A) preferred saliva-based genetic/epigenetic testing over blood (6%, 13/228), spinal fluid (1%, 3/228), and urine (1%, 2/228). These findings did not differ between groups (ASD vs. DD, p = 0.83; ASD vs. TD, p = 0.45; TD vs. DD, p = 0.69), supported by the non-significant impact of diagnosis on the overall model, (F(2,243) = 0.97, p = 0.38). A significant effect of parent age (F(1,243) = 4.62, p = 0.03) and consulting with a doctor to learn about genetics (F(1,243) = 5.42, p = 0.02) was found for responses indicating blood as the preferred biofluid.

## Discussion

This study of parental perspectives toward genetic and epigenetic research in ASD describes the views of parents whose children participated in a larger epigenetic study. The majority of parents had positive perceptions toward genetic/epigenetic research in ASD. Most parents indicated that “helping other children in the future” (85%) was the main reason for participation, suggesting altruism is a strong incentive in ASD molecular research.

Parents in this study indicated familiarity with both genetics and epigenetics, mainly learning this information by consulting with a doctor or using the internet. Despite increasing public awareness about molecular science and self-reported familiarity with genetics and epigenetics, when asked to indicate scientific differences between genetics and epigenetics, only 19% of parents demonstrated a clear understanding. This is consistent with previous studies examining public knowledge of genetics (Kaphingst et al. 2012; Morren et al. 2007). The lack of clear understanding did not appear to impact parents’ interest in a molecular test for ASD. Most parents desired a diagnostic test for ASD harnessing molecular technology, and the majority desired that all genetic/epigenetic results be reported, regardless of their implications for health and disease. The dichotomy between parental knowledge and parental desire for full genomic results underscores the necessity that any genetic/epigenetic test for ASD be ordered and interpreted in consultation with a health care provider.

Concerns expressed by parents with regards to genetic/epigenetic testing are similar to findings of other public opinion studies in the genetic and genomic literature, including concerns about protection of privacy, confidentiality, and restrictions on outside access to genetic/epigenetic results. The Genetic Information Nondiscrimination Act (GINA) was initiated to alleviate concerns about health insurers and employers obtaining access to genetic information and to protect individuals from discrimination using genetic risk profiles. GINA does not include life and disability insurance companies, and at this time, it does not explicitly cover epigenetic testing (NIH 2018; Rothstein 2018). GINA and state genetic non-discrimination laws should be amended to account for implementation of epigenetics in the clinic (Rothstein et al. 2009). Given the wide range of abilities exhibited by individuals with ASD as a function of the heterogeneity of the diagnosis, it would be difficult for insurance providers to place restrictions using epigenetic risk information. However, concerns about genetic/epigenetic information preventing families from obtaining insurance coverage may present a barrier to families seeking a molecular test.

Many parents indicated interest in a full return of results (regardless of medical implications). This finding deserves further exploration to better understand parental motivation for receipt of all results. To date, epigenetic testing is a novel approach, yet in time epigenetic test results may yield information about many adult-onset conditions for screening or diagnostic purposes. A long held practice in the field of genetics in healthcare is to avoid testing minors for adult onset conditions (Borry et al. 2006; Fallat et al. 2013; Lucassen et al. 2010; Shkedi-Rafid et al. 2015). This practice is based on the premise that it is an individual’s right to make an informed and autonomous decision as an adult to receive information about adult-onset conditions (Berkman and Hull 2014; Wolf et al. 2013). Much has been discussed about using the ‘best interest’ standard and the ‘harm’ principle when parents make health related decisions for their children (Birchley 2016; Diekema 2004, 2011; Pope 2011). The best interest standard suggests that decisions should be made considering what is best for the child, while the harm principle states that only actions that create harm to others should be prevented. More work needs to be done to understand potential harmful outcomes of parents receiving epigenetic results that later may indicate risk for various conditions.

Moreover, it is important to consider the familial impacts of these results when determining what is in the best interests of a child, given that genes among relatives are shared (Geelen et al. 2011). Considering that a child’s genome overlaps significantly with that of their parents’, it is possible that an indirect benefit of obtaining all genetic/epigenetic results is the ability to learn about possible genetic risks for parents as well (Green et al. 2013). Learning about a child’s medical future may have some benefit to families, but more research is needed to further understand the utility of parents obtaining all genetic/epigenetic results (Wilfond and Ross 2008).

Parents in this study desired that a molecular test for ASD be available earlier in development (12 months or younger) than current behavioral approaches (18 months or older). This finding may be explained by an increasing public awareness of childhood ASD prevalence, as well as evidence that early intervention can improve developmental outcomes for children with ASD (Dawson et al. 2010; Eldevik et al. 2009; Peters-Scheffer et al. 2011). In addition, recent publicity about limitations of behavioral screening assessments (Siu et al. 2016), and parental frustrations with the influence of situational variables on the diagnostic process (e.g., child’s mood, environmental variables) may drive requests for an objective molecular test. It is important to consider that approximately 75% of all parents in the study had a at least some college education, although there were no differences in education level between the ASD group and the DD and TD groups. Our study indicates that education level may influence the type of results parents wish to receive (e.g., only results associated with a disease) and the age at which they wish to receive results (e.g., at birth, or at 12 months). Highly educated parents may be more comfortable with ambiguous results or un-actionable results because they plan to rely on their own background knowledge for interpretation. Thus, the parent attitudes reported in the present study (containing 75% of parents with some college education) may not be universally generalizable.

Surprisingly, overall interest in ASD testing and many of the parental perspectives reported in the current study were not dependent on child developmental status (ASD, DD, or TD). The TD group endorsed value in molecular ASD testing, despite the fact that these parents had no children with ASD. This may result from increasing public awareness of ASD prevalence and the importance of early detection. Differences among parent groups were limited to: (1) parents of children with ASD felt more personal value in research and reported more interest in “helping their own children”; (2) parents of children with ASD had the most awareness of genetic/epigenetic information prior to participating in research; and (3) parents of children with ASD indicated more concerns about insurance related to genetic/epigenetic testing. Overall, most parents were interested in a molecular test for ASD regardless of their child’s developmental status.

### Clinical Implications

Molecular technology has the potential to address clinical need and parental preference by providing objective, biological evidence of ASD at an earlier age than behavioral assessments currently available in the diagnostic process. Results of this study suggest that saliva may be an ideal biofluid for ASD testing, given parents’ strong preference for this non-invasive approach. Development of a salivary test in children under 12-months of age would satisfy desires of community stakeholders while providing opportunity for early intervention. Early intervention programs are considered effective for children with ASD younger than 3 years and as early as 18 months. Some studies even suggest that parent-focused early intervention programs can be appropriate for children with ASD as young as 12 months of age (Dawson and Bernier 2013; Dawson et al. 2010). Currently, there are no ASD therapies for children under 12 months. This may be attributed, in-part, to the fact that there are no available tools to accurately diagnose children with ASD at such a young age. If this were possible, it could provide opportunities to develop new interventions. At the very least, it would provide more time to set up intervention services, which typically take months (or even years) to institute. Initiating interventions at the earliest possible age may have a profound impact on the developmental trajectory of children with ASD.

Considering parents’ overwhelming interest in test results, but lack of clear scientific understanding, it will be imperative that any molecular test for ASD be reviewed in consultation with a health professional. Resources for parents, such as genetic counseling, information about advocacy organizations, and parent support programs, should be offered along with testing results to guide and support parents through the diagnostic process.

There are several ethical, policy, and social challenges that will need to be addressed surrounding genetic/epigenetic testing for ASD. It is important to consider the potential impacts that epigenetic results may have when identifying an asymptomatic child as high risk; the social implications and stigma associated with releasing this information to parents, schools and health professionals; and the policy implications surrounding treatment and patient management following a child being identified as high risk for ASD. Expansion of epigenetic testing to the prenatal setting would require careful ethical considerations and specific protections for the rights of the unborn child. Lastly, as epigenetic research advances and the application of molecular technology expands, it will become increasingly likely that molecular results may yield insights about adult-onset conditions (or at least the risk for adult conditions). It will therefore be imperative that clinicians, scientists, legal experts, and the public work together to develop a responsible ethical approach to delivering epigenetic results and protecting those results in a manner that prioritizes individual patient interests. For adult onset conditions, in particular, this will require careful protection of electronic medical records and coordinated efforts between pediatric and adult physicians.

### Limitations

This study involved parents from a limited geographical region (clinics affiliated with academic medical centers in New York and Pennsylvania). It is possible that parental preferences may vary across different regions of the United States, and a larger survey might yield different insights. Although comparisons among parents of children with ASD, TD, and DD represent a novel approach in the current study, differences among ASD and DD groups should be interpreted with caution given the small sample size of the DD cohort (n = 39). Significant findings related to race should also be interpreted with caution given the small sample size of individual race categories. This study included parents who agreed to participate in genomic ASD research; therefore, the results may reflect a positive bias toward molecular testing and may underestimate parental concerns about genetic/epigenetic tests. However, participation rates in the genomic study generally exceeded 90%, suggesting that fewer than 10% of parental opinions are excluded from the current dataset.

## Conclusions

Recent advances in biomedical research have demonstrated the potential for molecular testing to provide an early, objective, and accurate adjunct to childhood ASD diagnosis. This study demonstrates high levels of parent interest in such a tool, regardless of child developmental status. Though parents voiced few concerns about epigenetic/genetic testing, review of any molecular results with a trained healthcare professional will be crucial, given the implications of an ASD diagnosis and the complex nature of epigenetic/genetic measures.

## References

[CR1] Abraham M, Othman A, Shahrouri M, Mustafa E (2014). Factors influencing public participation in biobanking. European Journal of Human Genetics.

[CR2] Anagnostou E, Zwaigenbaum L, Szatmari P, Fombonne E, Fernandez BA, Woodbury-Smith M, Scherer SW (2014). Autism spectrum disorder: Advances in evidence-based practice. CMAJ.

[CR3] Bates BR, Templeton A, Achter PJ, Harris TM, Condit CM (2003). What does “a gene for heart disease” mean? A focus group study of public understandings of genetic risk factors. American Journal of Medical Genetics Part A.

[CR4] Berkman BE, Hull SC (2014). The “right not to know” in the genomic era: time to break from tradition?. The American Journal of Bioethics.

[CR5] Beskow LM, Dean E (2008). Informed consent for biorepositories: assessing prospective participants’ understanding and opinions. Cancer Epidemiology, Biomarkers & Prevention.

[CR6] Biesecker LG, Mullikin JC, Facio FM, Turner C, Cherukuri PF, Blakesley RW, Green ED (2009). The ClinSeq Project: Piloting large-scale genome sequencing for research in genomic medicine. Genome Research.

[CR7] Birchley G (2016). Harm is all you need? Best interests and disputes about parental decision-making. Journal of Medical Ethics.

[CR8] Borry P, Stultiens L, Nys H, Cassiman JJ, Dierickx K (2006). Presymptomatic and predictive genetic testing in minors: A systematic review of guidelines and position papers. Clinical Genetics.

[CR9] Carter MT, Scherer SW (2013). Autism spectrum disorder in the genetics clinic: A review. Clinical Genetics.

[CR10] Catz DS, Green NS, Tobin JN, Lloyd-Puryear MA, Kyler P, Umemoto A, Wolman F (2005). Attitudes about genetics in underserved, culturally diverse populations. Community Genetics.

[CR11] Chokoshvili D, Belmans C, Poncelet R, Sanders S, Vaes D, Vears D, Borry P (2017). Public views on genetics and genetic testing: A survey of the general public in Belgium. Genetic Testing and Molecular Biomarkers.

[CR12] Dawson G, Bernier R (2013). A quarter century of progress on the early detection and treatment of autism spectrum disorder. Development and Psychopathology.

[CR13] Dawson G, Rogers S, Munson J, Smith M, Winter J, Greenson J, Varley J (2010). Randomized, controlled trial of an intervention for toddlers with autism: The early start denver model. Pediatrics.

[CR14] Diekema D (2004). Parental refusals of medical treatment: The harm principle as threshold for state intervention. Theoretical Medicine and Bioethics.

[CR15] Diekema D (2011). Revisiting the best interest standard: Uses and misuses. The Journal of Clinical Ethics.

[CR16] Eldevik S, Hastings RP, Hughes JC, Jahr E, Eikeseth S, Cross S (2009). Meta-analysis of early intensive behavioral intervention for children with autism. Journal of Clinical Child & Adolescent Psychology.

[CR17] Facio FM, Brooks S, Loewenstein J, Green S, Biesecker LG, Biesecker BB (2011). Motivators for participation in a whole-genome sequencing study: Implications for translational genomics research. European Journal of Human Genetics.

[CR18] Fallat M, Katz A, Mercurio M, Moon M, Okun A, Webb S, Weise K (2013). Ethical and policy issues in genetic testing and screening of Children. Pediatrics.

[CR19] Fischbach RL, Harris MJ, Ballan MS, Fischbach GD, Link BG (2016). Is there concordance in attitudes and beliefs between parents and scientists about autism spectrum disorder?. Autism.

[CR20] Geelen E, Van Hoyweghen I, Doevendans PA, Marcelis CL, Horstman K (2011). Constructing “best interests”: Genetic testing of children in families with hypertrophic cardiomyopathy. American Journal of Medical Genetics Part A.

[CR21] Godard B, Marshall J, Laberge C (2007). Community engagement in genetic research: Results of the first public consultation for the Quebec CARTaGENE project. Community Genetics.

[CR22] Gollust SE, Gordon ES, Zayac C, Griffin G, Christman MF, Pyeritz RE, Bernhardt BA (2012). Motivations and perceptions of early adopters of personalized genomics: Perspectives from research participants. Public Health Genomics.

[CR23] Goodacre JA, Levitt M, Weiner K (2005). Gene Week: A novel way of consulting the public. Public Understanding of Science.

[CR24] Green RC, Berg JS, Grody WW, Kalia SS, Korf BR, Martin CL, Ormond KE (2013). ACMG recommendations for reporting of incidental findings in clinical exome and genome sequencing. Genetics in Medicine.

[CR25] Haga SB, Zhao JQ (2013). Stakeholder views on returning research results. Advances in Genetics.

[CR26] Harris ED, Ziniel SI, Amatruda JG, Clinton CM, Savage SK, Taylor PL, Holm IA (2012). The beliefs, motivations, and expectations of parents who have enrolled their children in a genetic biorepository. Genetics in Medicine.

[CR27] Henneman L, Vermeulen E, van El CG, Claassen L, Timmermans DRM, Cornel MC (2012). Public attitudes towards genetic testing revisited: Comparing opinions between 2002 and 2010. European Journal Of Human Genetics.

[CR28] Hicks SD, Ignacio C, Gentile K, Middleton FA (2016). Salivary miRNA profiles identify children with autism spectrum disorder, correlate with adaptive behavior, and implicate ASD candidate genes involved in neurodevelopment. BMC Pediatrics.

[CR29] Hicks SD, Middleton FA (2016). A comparative review of microRNA expression patterns in Autism Spectrum Disorder. Front Psychiatry.

[CR30] Hicks SD, Uhlig R, Afshari P, Williams J, Chroneos M, Tierney-Aves C, Middleton FA (2018). Oral microbiome activity in children with autism spectrum disorder. Autism Research.

[CR31] Hull SC, Sharp RR, Botkin JR, Brown M, Hughes M, Sugarman J, Wilfond BS (2008). Patients’ views on identifiability of samples and informed consent for genetic research. The American Journal of Bioethics.

[CR32] Johannessen J, Naerland T, Bloss C, Rietschel M, Strohmaier J, Gjevik E, Andreassen OA (2016). Parents’ attitudes toward genetic research in autism spectrum disorder. Psychiatric Genetics.

[CR33] Johnson CP, Myers SM (2007). Identification and evaluation of children with autism spectrum disorders. Pediatrics.

[CR34] Kaphingst KA, Facio FM, Cheng MR, Brooks S, Eidem H, Linn A, Biesecker LG (2012). Effects of informed consent for individual genome sequencing on relevant knowledge. Clinical Genetics.

[CR35] Kaufman DJ, Murphy-Bollinger J, Scott J, Hudson KL (2009). Public opinion about the importance of privacy in biobank research. American Journal of Human Genetics.

[CR36] Kettis-Lindblad A, Ring L, Viberth E, Hansson MG (2006). Genetic research and donation of tissue samples to biobanks. What do potential sample donors in the Swedish general public think?. European Journal of Public Health.

[CR37] Lai MC, Lombardo MV, Baron-Cohen S (2014). Autism. Lancet.

[CR38] Landgrave-Gómez J, Mercado-Gómez O, Guevara-Guzmán R (2015). Epigenetic mechanisms in neurological and neurodegenerative diseases. Frontiers in Cellular Neuroscience.

[CR39] Lanie AD, Jayaratne TE, Sheldon JP, Kardia SL, Anderson ES, Feldbaum M, Petty EM (2004). Exploring the public understanding of basic genetic concepts. Journal of Genetic Counseling.

[CR40] Lea DH, Kaphingst KA, Bowen D, Lipkus I, Hadley DW (2011). Communicating genetic and genomic information: Health literacy and numeracy considerations. Public Health Genomics.

[CR41] Lee IH, Kang HY, Suh HS, Lee S, Oh ES, Jeong H (2018). Awareness and attitude of the public toward personalized medicine in Korea. PLoS ONE.

[CR42] Lemke AA, Wolf WA, Hebert-Beirne J, Smith ME (2010). Public and biobank participant attitudes toward genetic research participation and data sharing. Public Health Genomics.

[CR43] Loke YJ, Hannan AJ, Craig JM (2015). The role of epigenetic change in autism spectrum disorders. Frontiers in Neurology.

[CR44] Lucassen, A., Clancy, T., Montgomery, J., Clarke, A., Hall, A., Fryer, A., … Parker, M. (2010). Report on the genetic testing of children 2010. British Society for Human Genetics. Retrieved from https://www.bsgm.org.uk/media/678741/gtoc_booklet_final_new.pdf.

[CR45] Ludman EJ, Fullerton SM, Spangler L, Trinidad SB, Fujii MM, Jarvik GP, Burke W (2010). Glad you asked: Participants’ opinions of re-consent for dbGap data submission. Journal of Empirical Research on Human Research Ethics.

[CR46] McGuire AL, Hamilton JA, Lunstroth R, McCullough LB, Goldman A (2008). DNA data sharing: Research participants’ perspectives. Genetics in Medicine.

[CR47] Miller JD (2004). Public understanding of, and attitudes toward, scientific research: What we know and what we need to know. Public Understanding of Science.

[CR48] Molster C, Charles T, Samanek A, O’Leary P (2009). Australian study on public knowledge of human genetics and health. Public Health Genomics.

[CR49] Morren M, Rijken M, Baanders AN, Bensing J (2007). Perceived genetic knowledge, attitudes towards genetic testing, and the relationship between these among patients with a chronic disease. Patient Education and Counseling.

[CR50] NIH, G. H. R. (2018). What is genetic discrimination? *Genetics home reference*. Retrieved from https://ghr.nlm.nih.gov/primer/testing/discrimination.

[CR51] Oberg JA, Glade Bender JL, Cohn EG, Morris M, Ruiz J, Chung WK, Levine JM (2015). Overcoming challenges to meaningful informed consent for whole genome sequencing in pediatric cancer research. Pediatric Blood & Cancer.

[CR52] Olson JE, Ryu E, Johnson KJ, Koenig BA, Maschke KJ, Morrisette JA, Cerhan JR (2013). The mayo clinic Biobank: A building block for individualized medicine. Mayo Clinic Proceedings.

[CR53] Ormond KE, Cirino AL, Helenowski IB, Chisholm RL, Wolf WA (2009). Assessing the understanding of biobank participants. American Journal of Medical Genetics Part A.

[CR54] Pellicano E, Stears M (2011). Bridging autism, science and society: Moving toward an ethically informed approach to autism research. Autism Research.

[CR55] Peters-Scheffer N, Didden R, Korzilius H, Sturmey P (2011). A meta-analytic study on the effectiveness of comprehensive ABA-based early intervention programs for children with Autism Spectrum Disorders. Research in Autism Spectrum Disorders.

[CR56] Pope TM (2011). The best interest standard: Both guide and limit to medical decision making on behalf of incapacitated patients. The Journal of Clinical Ethics.

[CR57] Rahm AK, Wrenn M, Carroll NM, Feigelson HS (2013). Biobanking for research: A survey of patient population attitudes and understanding. Journal of Community Genetics.

[CR58] Reiff M, Bugos E, Giarelli E, Bernhardt BA, Spinner NB, Sankar PL, Mulchandani S (2017). “Set in stone” or “ray of hope”: Parents’ beliefs about cause and prognosis after genomic testing of children diagnosed with ASD. Journal of Autism and Developmental Disorders.

[CR59] Reiff M, Giarelli E, Bernhardt BA, Easley E, Spinner NB, Sankar PL, Mulchandani S (2015). Parents’ perceptions of the usefulness of chromosomal microarray analysis for children with autism spectrum disorders. Journal of Autism and Developmental Disorders.

[CR60] Rothstein MA (2018). GINA at ten and the future of genetic nondiscrimination law. Hastings Center Report.

[CR61] Rothstein MA, Cai Y, Marchant GE (2009). The ghost in our genes: Legal and ethical implications of epigenetics. Health Matrix Clevel.

[CR62] Sapp JC, Dong D, Stark C, Ivey LE, Hooker G, Biesecker LG, Biesecker BB (2014). Parental attitudes, values, and beliefs toward the return of results from exome sequencing in children. Clinical Genetics.

[CR63] Scheaffer, R. L., Mendenhall, W. III, Ott, R. L., & Gerow, K. G. (2011). *Elementary survey sampling*: Cengage Learning.

[CR64] Shkedi-Rafid S, Fenwick A, Dheensa S, Lucassen AM (2015). Genetic testing of children for adult-onset conditions: Opinions of the British adult population and implications for clinical practice. European Journal Of Human Genetics.

[CR65] Siu AL, Bibbins-Domingo K, Grossman DC, Baumann LC, Davidson KW, Ebell M, Kemper AR (2016). Screening for autism spectrum disorder in young children: US Preventive Services Task Force recommendation statement. JAMA.

[CR66] Trinidad SB, Fullerton SM, Bares JM, Jarvik GP, Larson EB, Burke W (2010). Genomic research and wide data sharing: Views of prospective participants. Genetics in Medicine.

[CR67] Wilfond B, Ross LF (2008). From genetics to genomics: Ethics, policy, and parental decision-making. Journal of Pediatric Psychology.

[CR68] Wolf SM, Annas GJ, Elias S (2013). Patient autonomy and incidental findings in clinical genomics. Science.

[CR69] Wong ML, Chia KS, Yam WM, Teodoro GR, Lau KW (2004). Willingness to donate blood samples for genetic research: A survey from a community in Singapore. Clinical Genetics.

[CR70] Xu L, Mitchell LC, Richman AR, Luo H, Jiang Y, Driggers AL, Floyd AE (2018). Parental knowledge and perceptions of pediatric genomic testing for autism spectrum disorders in rural settings. Advances in Neurodevelopmental Disorders.

